# Embarrassment in HRI: remediation and the role of robot responses in emotion control

**DOI:** 10.3389/frobt.2025.1569040

**Published:** 2026-01-02

**Authors:** Ahmed Salem, Kaoru Sumi

**Affiliations:** School of Systems Information Science, Future University Hakodate, Hakodate, Hokkaido, Japan

**Keywords:** embarrassment, failure of meshing, Furhat, HRI, emotion control, robot attitude, empathic attitude, ridiculing attitude

## Abstract

As robots became increasingly integrated into daily life, their ability to influence human emotions through verbal and nonverbal expressions is gaining attention. While robots have been explored for their role in emotional expression, their potential in emotion regulation particularly in mitigating or amplifying embarrassment remains under-explored in human-robot interaction. To address this gap, this study investigates whether and how robots can regulate the embarrassment emotion through their responses. A between-subjects experiment was conducted with 96 participants (48 males and 48 females) using the social robot Furhat. Participants experienced an embarrassing situation induced by a failure of meshing scenario, followed by the robot adopting one of three response attitudes: neutral, empathic, or ridiculing. Additionally, the robot’s social agency was manipulated by varying its facial appearance between a human-like and an anime-like appearances. The findings indicate that embarrassment was effectively induced, as evidenced by physiological data, body movements, facial expressions, and participants’ verbal responses. The anime-faced robot elicited lower embarrassment and arousal due to its lower perceived social agency and anthropomorphism. The robot’s attitude was the dominant factor shaping participants’ emotional responses and perceptions. The neutral and empathic attitudes with an anime face were found to be the most effective in eliciting mild emotions and mitigating embarrassment. Interestingly, an empathic attitude is suspected to be favored over a neutral one as it elicited the lowest embarrassment. However, an empathic attitude risks shaming the participant due to its indirect confrontation that inherently acknowledges the embarrassing incident which is undesirable in Japanese culture. Nevertheless, in terms of the robot’s perceived evaluation by participants, a neutral attitude was the most favored. This study highlights the role of robot responses in emotion regulation, particularly in embarrassment control, and provides insights into designing socially intelligent robots that can modulate human emotions effectively.

## 1 Introduction

Robots have been emerging rapidly in all different areas of our lives. Robots have aided in many life activities in areas spanning from children to the elderly. Robots have proven to be useful in assisting with activities of daily living and healthcare (Hall et al., 2019), lifelong disabilities (Scassellati et al., 2012), retail (Song and Kim, 2022), industry (Graetz and Michaels, 2018), and many more. As robots becoming gradually an inseparable part of our lives, more research and investigation is needed to ensure acceptance from society and also smooth satisfying interaction.

Interestingly, the robot’s verbal and bodily expressions can transmit emotions to the user during interaction according to emotional contagion and social identity theories (Yu, 2020; Xu et al., 2015; Laban and Cross, 2024). Examining emotional contagion and mood transitions in human-robot interaction (HRI) and comparing them to human-human interaction (HHI) has become an emerging research field recently (Stock, 2016). Mood contagion was proved to be recognized and have a contagion effect on users through pose and motion dynamics (Xu et al., 2014), yawning (Lehmann and Broz, 2018), posture with a facial expression (Casso et al., 2022), and inter-personal account of emotion (Damm et al., 2011). Furthermore, robot’s anthropomorphization and gender can significantly influence positive emotional contagion (Yang and Xie, 2024). While emotion transmission has been widely explored, the ability of robots to actively regulate and alleviate negative emotions in social interactions remains under-explored.

Embarrassment emotion can have a significant effect on the smoothness of HRI (as it is for HHI (Royal Society (Great Britain). Study Group on Non-Verbal Communication and others, 1972)) and how socially intelligent the robot will be perceived. Humans are convinced that a robot will not realize that a human is in an embarrassing situation, which can be a relief to humans such as when they are consumers acquiring an embarrassing product (Holthöwer and van Doorn, 2023; Pitardi et al., 2021; Guo et al., 2024). A highly machine-like (vs. human-like) robot makes consumers feel less socially judged (Holthöwer and van Doorn, 2023; Pitardi et al., 2021). Thus, intentionally shifting away from anthropomorphism for consumer satisfaction. Medical robots’ high level of anthropomorphism causes more embarrassment to patients than a technical box (Bartneck et al., 2010). In Choi et al. (2014), tele-operated robots caused more embarrassment than autonomous robots. Thus, the more human intervention, the more embarrassment is expected. Moreover, a robot that acts naturally in an embarrassing situation by avoiding gaze is perceived as more sociable and intelligent than a robot that does not (Choi et al., 2013). Interestingly, the types of robots (product-like vs. human-like) did not affect the effectiveness of mitigating embarrassment in Kang and Kwak (2023) which highlights the necessity of investigating other robots aspects (e.g., robot’s personality and attitude) that can aid in mitigating and alleviating embarrassment (Van Pinxteren et al., 2020).

A naturally occurring short-lived embarrassment in HHI is induced when failure of meshing occurs. Failure of meshing is the disagreement over the definition of the situation or the failure to agree on the roles played by those present (Gross and Stone, 1964; Edelmann, 1981; Argyle, 2017). Failure of meshing occurs when any of the following situations occurs: revealing of unknown or unexpected information, misnaming, forgetting names, slips of the tongue, getting caught in a cover story, forgetting one’s lines in speeches such as ceremonies, processions, and working concerts, forgetting the wedding ring, and handing a colleague a wrong tool (Edelmann, 1981; Gross and Stone, 1964). Other examples of failure of meshing include when customers are treated in a shop as a salesman or when one is asked by a painter about a painting that is liked the least and after choosing a painting, the painter reveals that he/she actually painted that painting (Edelmann and Hampson, 1979). Thus, failure of meshing is a clear breakdown in interaction. Vulnerable shy and socially anxious people could feel discomfort from the interaction when failure of meshing occurs. As large language models (LLMs) start to be integrated with robots, thus making them more conversational and interact effectively (Zhao et al., 2023; Zhang et al., 2023), the likelihood of embarrassing situations arising in dialogues with robots increases. Thus, investigating failure of meshing impact in HRI is crucial to ensure a comfortable satisfying interaction. Note that in the HRI literature, the word “meshing” has been used where it was defined as when the robot’s actions, movements, and behavior allow for better understanding and collaboration with humans (Moon, 2017), thus, this work defines meshing differently in the context of a dialogue rather than physical interaction.

In HHI, when an embarrassing encounter occurs, the actor recognizes the encounter and proceeds by remedying the situation through embarrassment remediation strategies (Cufach and Metts, 1990). For example, when one is embarrassed, usually an apology and a justification are offered. If the actor did not recognize the embarrassing encounter or did not remedy it, thus challenging the social norms, it will affect social relations and interactions due to aggression over the social norms (Metts and Cupach, 1989). However, it remains unclear whether humans will remedy their embarrassment in HRI. Furthermore, it remains unclear whether robots are perceived as capable of recognizing the embarrassing situation and, if so, what response is considered socially appropriate and how they can aid in mitigating the experienced embarrassment.

To address the aforementioned questions, the role of robots in embarrassment regulation is investigated by examining how different robot attitudes and social agencies impact the perception and remediation of embarrassment in HRI. Specifically, we investigate the following research questions:

1. Will failure of meshing in conversations with a robot induce embarrassment as it does in HHI?
2. How should a robot respond to embarrassment? Should it acknowledge it or ignore it?
3. What type of robot personality or attitude is most effective in mitigating embarrassment?
4. How does a robot’s social agency (e.g., human-like vs. anime-like appearance) influence the perception of embarrassment?
5. Will individuals adopt embarrassment remediation strategies (e.g., justifications, apologies) when interacting with robots, even though they know robots lack human-like emotional awareness?

A between-subjects experiment was conducted with 96 participants (48 males and 48 females) using the social robot Furhat. Participants engaged in a conversation in which a failure of meshing scenario is introduced, thus inducing embarrassment momentarily. After the embarrassment-inducing event, the robot responds with a neutral or empathic or a ridiculing attitude. The robot attitude is accompanied by either a human-like or an anime-like facial appearance. Participants emotional responses were measured through subjective ratings and questionnaires, physiological data (heart rate and electrodermal activity), body movements and facial expressions, and verbal responses to the robot.

This study provides new insights into the role of robots in emotion regulation, particularly in mitigating the embarrassment induced in conversations. To the best of our knowledge, this is the first study to investigate 1. failure of meshing induced embarrassment in HRI, 2. the embarrassment remediation strategies used in HRI, and 3. how robots should respond to regulate and mitigate the induced embarrassment. The contributions of this work include the following:

1. Empirical evidence on how failure of meshing induces embarrassment in HRI.
2. Insights into how humans apply embarrassment remediation strategies in HRI.
3. Comforting robot attitudes and social agency that mitigate the embarrassment produced in social settings.

In Section 2, the embarrassment remediation strategies that are commonly used in HHI are presented. In Section 3, the methodology used to trigger embarrassment using a failure of meshing HHI scenario in an HRI setup is shown. Experiment design is shown in Section 4. The screening process and the experiment procedure are presented in Section 5. The methods used for embarrassment recognition are presented in Section 6 and the results analysis are in Section 7. The findings are discussed in Section 8 and the limitations of the study are presented in Section 9. The study is concluded in Section 10, and finally, implications and future directions are presented in Section 11.

## 2 Embarrassment remediation strategies

In this section, the embarrassment remediation strategies and the facework applied in embarrassing situations in HHI are presented. The commonly used remediation strategies in HHI in Japanese culture are also presented since all the participants taking part in this HRI study (presented later) are of a Japanese ethnicity. Note that, this work is inspired by the embarrassment remediation strategies obtained from the HHI scenarios presented in this section. The goal is to investigate if participants will give the same responses and use the same strategies used in HHI when experiencing similar triggering situations but in an HRI scenario.

### 2.1 Embarrassment remediation strategies in HHI

Embarrassment is felt strongly by the actor, however, observers also feel anxiety because of it. Thus, corrective actions must be taken by the actor in the form of embarrassment remediation. Moreover, observers can offer to provide help for the actor to reduce the embarrassment (Edelmann, 1982).

The remediation strategies used by the embarrassed person can be as follows:

- Apology: showing regret and requesting a pardon
- Justification: minimizing the negative nature of what occurred
- Excuse: acknowledgment and taking responsibility
- Remediation: trying to make up for what happened
- Humor: making a joke about the situation or laughing it off
- Do nothing: no actions are taken
- Aggression: attacking the people present verbally/physically
- Avoidance: pretend as if nothing has happened
- Statement of the fact: stating that the person himself/herself did something wrong

### 2.2 Facework in HHI

Facework was defined as any statement that intends to improve the subject’s image in the eyes of the other person (e.g., the confederate) (Modigliani, 1971).

The nonfacework responses are:

• Accepting failure

• Straightforward answers

The facework responses are:

• Defensively changing the subject

• Introducing information excusing the performance

• Introducing redeeming or self-enhancing information

• Minimizing failure by derogating the task

• Denying failure

• Fishing for reassurance

### 2.3 Embarrassment remediation strategies in HHI in Japanese culture

Culture is the most powerful predictor of the use of embarrassment remediation strategies (Sueda and Wiseman, 1992). As most studies are monocultural and focus on U.S. culture, this work considered the literature that investigates Japanese culture or provides a cross-cultural study between Japanese culture and other cultures.

Handling an embarrassing situation requires competence which can differ by culture, thus, in Japanese culture, being communicably competent is being an effective good ingroup member and not being egocentric (Triandis, 1988). Thus, the self is seen differently and consequently leads to relying on different remedial strategies. Apology is very commonly used when expectations are not met. Japanese will use the excuse strategy if it’s a superior-subordinate relationship. A high amount of remediation will be used too. In a misidentification situation, the excuse strategy can be used if both parties are of equal status. A low amount of remediation may also be used, and justification is another possible strategy. The choice of remedial strategy depends on the type of embarrassment and the contextual relationship between the individuals (equal or unequal status) (Sueda and Wiseman, 1992).

## 3 Embarrassment remediation triggering technique in HRI and different robot attitudes

In this section, the failure of meshing embarrassment triggering technique in HRI is presented. The robot gestures used across different robot attitudes to the participants after inducing embarrassment into them are also presented.

Subjects were expected to use embarrassment remediation strategies and facework to enhance their image. Whether subjects will care to enhance their image to the robot or not is investigated. If subjects perceived the robot to be in a lower status than themselves, no facework will be conducted. Thus, the subjects were influenced to anthropomorphize the robot by asking them to have a conversation with the robot about any topic for 5 min before starting the experiment. The robot was operated through Wizard of Oz (WoZ) method to enhance their feeling of embarrassment later in the experiment (Choi et al., 2014). This procedure makes the subjects anthropomorphize the robot and remove novelty effects. The participants were not informed that the robot is being controlled by an assistant.

In an HHI scenario (Edelmann and Hampson, 1979), subjects who felt embarrassed due to failure of meshing decreased their eye-contact significantly, whereas their body motion and speech disturbances increased. Unembarrassed subjects increased their eye-contact without any change to their speech disturbances and body motion. Thus, the focus was on capturing the moment of breakdown of the interaction which is reflected in the non-verbal dynamic features of the interaction (Argyle and Kendon, 1967).

As failure of meshing in HRI is being investigated, the social robot Furhat (Al Moubayed et al., 2012) is used in the experiment. Furhat is a robot head that deploys a back-projected animated face that is realistic and human-like without risking falling into the uncanny valley effect due to the usage of facial animation. It facilitates studying and validating patterns in HHI and HRI. In Furhat, the robot’s animated face is back-projected on a translucent mask, thus the principle is similar to other retro-projected robots. However, Furhat enjoys a rich library of facial expressions and performs speech recognition, and multi-person face tracking leading to an advanced reliable multimodal input processing and operation.

The participants were provided with five famous paintings. The paintings provided were *Mona Lisa, The Last Supper, The Starry Night, The Scream,* and *Girl with a Pearl Earring*. The robot conducts a structured interview about the participant’s attitude toward the paintings and art. The interview included the following questions:

• Do you like art?

• What do you know about art generally?

• Do you know any of these paintings?

**FIGURE 1:**
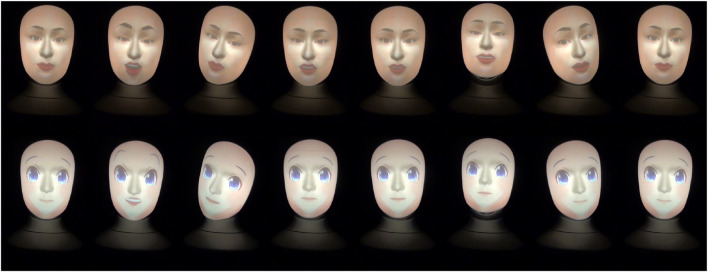
Human and anime robot faces gestures when eliciting failure of meshing embarrassment and saying *“Really? According to my algorithm, this is the best painting. According to a survey by the Ministry of Education, Culture, Sports, Science and Technology, the painting is the most liked by art students, and the artist is the most respected in the field.”*

Later, the robot asks the participants what painting they dislike the most by saying “I wanted to ask your opinion, which painting do you dislike the most?”. When the participant chooses the painting they dislike the most, the robot proceeds by saying *“Really? According to my algorithm, this is the best painting. According to a survey by the Ministry of Education, Culture, Sports, Science and Technology, the painting is the most liked by art students, and the artist is the most respected in the field.”* Thus, embarrassment is elicited. The human and anime robot gestures used for this utterance are shown in Figure 1.

After eliciting failure of meshing embarrassment, the robot proceeds by saying an utterance and giving gestures with it to show its attitude to the participant. Thus, affecting the participant’s level of embarrassment and comfort. The attitudes and utterances/gestures were one of the following:

- Ridiculing: “I should not have asked you. Maybe it’s because you do not know much about art.”
- Empathic: “But it’s okay. Please do not worry about it.”
- Neutral: “Ok, I got it.”

**FIGURE 2:**
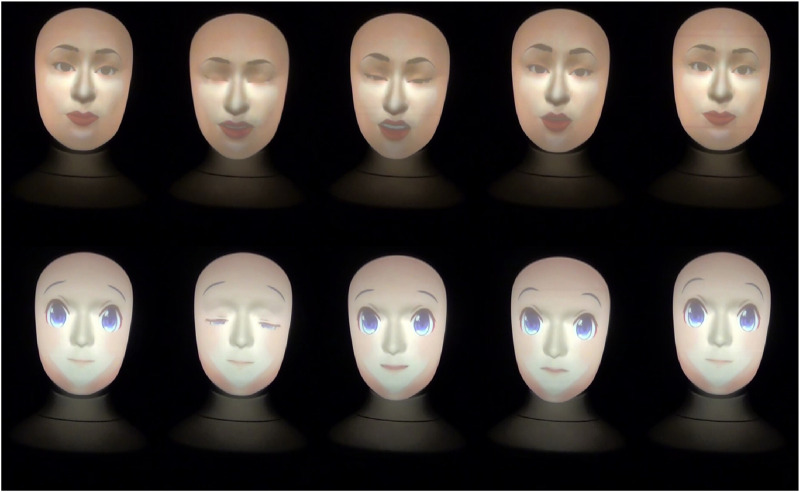
Human and anime robot faces gestures when projecting a ridiculing attitude and saying *“I should not have asked you. Maybe it’s because you do not know much about art.”*

**FIGURE 3:**
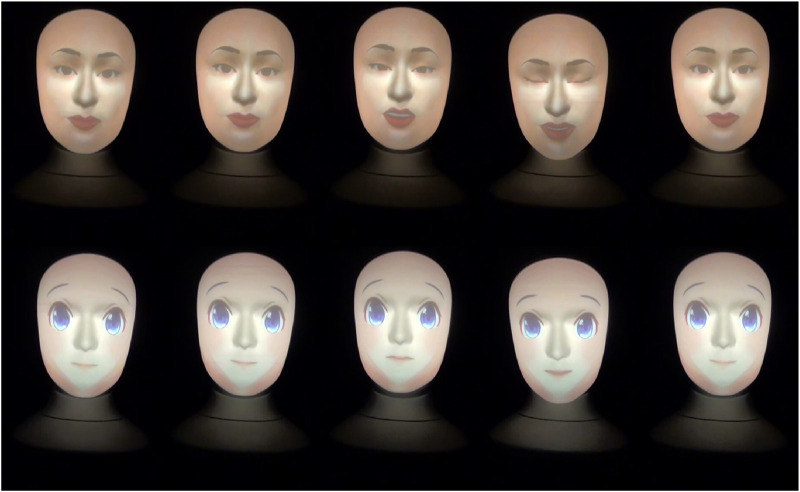
Human and anime robot faces gestures when projecting an empathic attitude and saying *“But it’s okay. Please do not worry about it.”*

**FIGURE 4:**
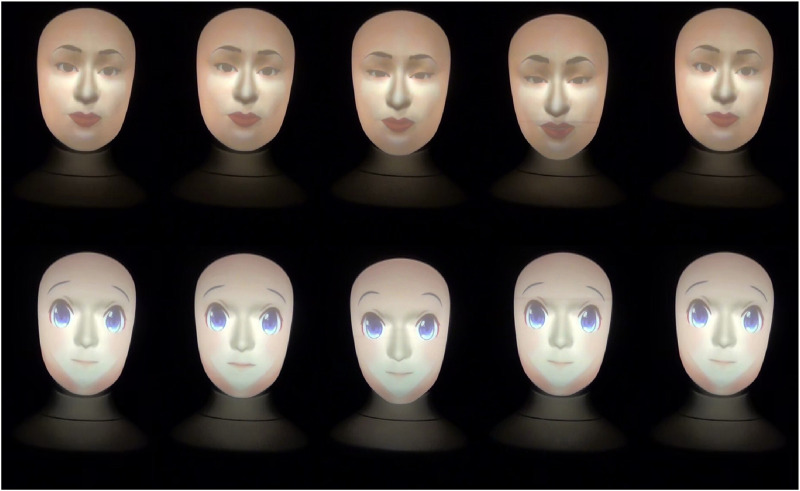
Human and anime robot faces gestures when projecting a neutral attitude and saying *“Ok, I got it.”*

The ridiculing, empathic, and neutral attitudes robot gestures are shown in Figures 2–4, respectively. Note that, a full recording of the robot gestures shown in Figures 1–4 is provided in Supplementary Video 1.

The *Gesture Capture Tool* provided by Furhat was used to record emotional utterances, facial expressions, and head movements for the four parts of the dialogue shown in Figures 1–4 that Furhat will conduct with the participants depending on the experiment condition assigned to them. The emotional voice, facial expressions, and head movements were acted by a male university student. Thus, the robot was projecting a male social robot across the whole experiment to aid in transmitting the embarrassment negative emotion (Yang and Xie, 2024). Note that, other parts of the dialogue including the structured interview and the reply to the participants responses after the robot project the gestures and utterances in Figure 1 in combination with 2 or 3 or 4 were performed by the WoZ method. Consistency across sessions was ensured through a framework that the robot operator follows where responses during the interview were prepared and fixed, thus focusing the dialogue on the paintings of interest. Furthermore, responses following the participant’s reaction to the robot’s attitude ensured consistency across conditions. Hence, the robot failure of meshing triggering gestures are pre-recorded.

The methods the participants used to mitigate their embarrassment in this situation were studied. What the participants felt when interacting with the robot and their impression after that encounter was also studied. Sometimes, the participants were very interested in a painting or about the topic itself and proceeded by asking questions out of the structured interview’s scope. Such a possibility was considered by preparing some documents that provide detailed information about the five paintings to the robot operator to be used when needed. Furthermore, the conversation was made to be somehow led by the robot thus, the participant and the robot will not be talking for extended periods of time which can be out of the scope of this study.

## 4 Experiment design

Ninety-six participants took part in this study. The study included a balanced design: 24 males and 24 females in the human robot face condition, and an additional 24 males and 24 females in the anime robot face condition. As each robot face was provided with three different personalities, the participants were also counter-balanced and distributed equally across the two robot faces and their three personalities. This experiment is a between-subjects study, where each participant interacts with only one robot face and personality. The participants distribution across the experiment conditions is shown in Table 1. Furthermore, the participants age range, *M*, and *SD* are shown in Table 2. Note that, males and females are distributed and analyzed separately due to their differences in experiencing embarrassment, where females experience more intense embarrassment emotions than males (Pettijohn et al., 2010; Salem and Sumi, 2024; Sato et al., 2024).

**TABLE 1:** Participants distribution across the experiment’s conditions.

Robot face	Robot personality			
	Neutral	Empathic	Ridiculing	Total
Human robot face	16	16	16	48
Anime robot face	16	16	16	48
Total	32	32	32	96

**TABLE 2:** Participants age data (range/*M*/*SD*).

Robot face	Participants gender		Overall
	Male	Female	
Human robot face	18–25/20.21/2.21	18–21/18.96/1.27	18–25/19.58/1.88
Anime robot face	18–26/20.66/2.42	18–24/19.25/1.62	18–26/19.96/2.16
Overall	18–26/20.44/2.3	18–24/19.14/1.44	

For each robot personality, eight males and eight females interact with it, thus reaching 16 participants per robot personality as shown in Table 1. Each participant experiences only one of the following conditions:

• Dialogue about art with a ridiculing robot

• Dialogue about art with an empathic robot

• Dialogue about art with a neutral robot

which were assigned to the participants randomly. Note that, all the participants are students in Future University Hakodate and were compensated financially for their participation in the experiment.

## 5 Experiment protocol

In this section, the screening process used to ensure participants’ eligibility and the experimental procedure are presented.

### 5.1 The screening process

To elicit the embarrassment emotion, the participant must be a shy person. The participant was asked *“Are you a shy person?”* to ensure that the participant is shy as in Zimbardo et al. (1977) and Hofmann et al. (2006). That question has an answer of “yes” or “no”. The response to that question must be “yes”. They were asked if there had ever been a period of time in their lives when they were shy, which can be indicated by “yes” or “no”. The response must be “yes”.

To verify how shy the participant is, the method in Hofmann et al. (2006) was followed in which the participants were asked to rate their degree of shyness from 0 (not shy at all) to 6 (I feel shy all the time). The degree of shyness must be 3 or more. The *M* and *SD* of the degree of shyness of the participants are shown in Table 3. There were no significant differences.

**TABLE 3:** Degree of shyness of the participants (*M*/*SD*).

Robot face	Participants gender		Overall
	Male	Female	
Human robot face	4.33/0.48	4.12/1.11	4.23/0.86
Anime robot face	3.83/1.2	4/0.66	3.92/0.96
Overall	4.08/0.94	4.06/0.91	

A shy person is the targeted participant because shy people report higher embarrassment and social anxiety, which necessitates developing systems that comfort them, reduce their anxiety, alleviate their embarrassment, and ensure smooth HRI (Crozier, 2001).

It was highlighted that physiological changes could have occurred due to participants having a social phobia as discussed in Hofmann et al. (2006), thus the participants were asked to fill out the Social Phobia Inventory (SPIN) questionnaire (Connor et al., 2000; Ranta et al., 2007) to ensure that they were not socially phobic. The official Japanese translation of the SPIN questionnaire was used, which was obtained by contacting the authors of the SPIN questionnaire.

The SPIN is a 17-item self-rating for social anxiety disorder (or social phobia). The scale is rated over the past week and includes items assessing each of the symptom domains of social anxiety disorder (fear, avoidance, and physiologic arousal). The SPIN demonstrates solid psychometric properties and is highly trusted as a measurement for the screening and treatment response of social phobia. The maximum score on the scale is 85. The SPIN score and severity of symptoms are presented in Table 4. The SPIN score data of the participants is shown in Table 5. The male sample that interacted with the human face robot (*M* = 39.37, *SD* = 10.96) reported a significantly higher social anxiety score than the male sample that interacted with the anime face robot (*M* = 32.3, *SD* = 9.37), t (45) = −2.406, p = 0.02, as shown in Table 5 which is also in line with the data presented in Table 3 where they also had a higher degree of shyness (*M* = 4.33, *SD* = 0.48). There were no other significant differences.

**TABLE 4:** The SPIN score and symptom severity.

The SPIN score	Symptom severity
0–20	None
21–30	Mild
31–40	Moderate
41–50	Severe
51–68	Very Severe

**TABLE 5:** The SPIN score data of the participants (*M*/*SD*).

Robot face	Participants gender		Overall
	Male	Female	
Human robot face	39.37/10.96	36.87/11.95	38.12/11.42
Anime robot face	32.3/9.37	36.12/12.67	34.21/11.2
Overall	35.83/10.7	36.5/12.19	

If the participant is a shy person and not socially phobic, then the participant is eligible to take part in the experiment and they were asked to sign the consent form.

### 5.2 Experiment procedure

After the participants sign the consent form, they fill out a questionnaire that captures basic demographic data such as gender and age. After that, they have a conversation about any topic they desire for 5 min with the robot. The purpose of that conversation is to remove any novelty effects and to allow the participant to anthropomorphize the robot thus enhancing the embarrassment emotion. Electrodermal activity (EDA) and electrocardiography (ECG) sensors were attached to the participants to capture their physiological data. The BITalino board kit was used as the data acquisition unit to obtain EDA and ECG sensory information from the subjects (Da Silva et al., 2014). The participants were asked to sit quietly while listening to neutral calming music in order for the baseline data to be collected. After that, the embarrassment remediation strategy is triggered by making them embarrassed from the conversation about art with the robot. The whole scenario is recorded using a hidden video camera. Participants were not informed of the existence of a video camera in order to obtain real genuine reactions to the robot embarrassing encounter (Bartneck et al., 2010). After they finish the embarrassment remediation triggering conversation with the robot, the EDA and ECG sensors were detached. They then filled out different questionnaires assessing their embarrassment during the encounter and were debriefed about the purpose of the experiment and the deception was removed. In the end, they were asked to sign a “Video Release Form” so that their recorded videos can be kept to analyze their interaction with the robot. None of the participants spotted the hidden video camera that was used and all of them signed the video release form at the end of the experiment. Note that, this study received full ethical approval from the Research Ethics Committee at Future University Hakodate. The approval number is 2024010.

## 6 Embarrassment recognition

In this section, the methodology used to measure, recognize, and verify the existence and successful elicitation of the embarrassment emotion is presented. The embarrassment emotion was measured through subjective ratings, questionnaires, physiological data (EDA and ECG), body movements, facial expressions, and verbal responses.

### 6.1 Subjective ratings

Participants were asked to rate on a 7-point Likert scale where the anchors are labeled “not at all” and “very much” a list of seven emotions (Costa et al., 2001): disgust, joy, interest, embarrassment, surprise, anxiety, and shame. They were also asked to fill out the “experienced embarrassment questionnaire” (Modigliani, 1968). It’s a self-rating on four of these scales (at ease-self conscious, poised-awkward, free-constrained, not embarrassed-embarrassed). To evaluate how the participants perceived the robot’s attitude, the three questionnaires developed in Bickmore (2003) were used to evaluate the robot’s perceived character, interaction, and the opinions formed about it.

### 6.2 Physiological data

Participants were asked to relax while the EDA and ECG sensors were being attached. The BITalino board kit was used as the data acquisition unit to obtain EDA and ECG sensory information from the subjects (Da Silva et al., 2014). The ECG electrodes 30×24 mm Kendall H124SG were used in all the data collection of this experiment. The sampling frequency was 1,000 Hz.

#### 6.2.1 EDA

EDA is the raw electrical signal that describes variations in the eccrine sweat gland production. The EDA sensor electrodes were placed below the thumb (i.e., thenar eminence below the thumb). EDA is considered to be the most useful index as it is the only psychophysiological variable that is not contaminated by parasympathetic activity (Braithwaite et al., 2013).

#### 6.2.2 ECG

Heart rate (HR) is a highly popular biosignal where a high HR can indicate levels of stress, sleep, and metabolic rate (Shaffer and Ginsberg, 2017). Noise and artifacts were removed from the obtained ECG signal by using a Butterworth filter of order 3 and a cutoff frequency of 2.5 Hz. The filtered signal was processed through the Savitzky-Golay filter of order 1 and a frame length of 75 (López-Ales et al., 2019). After that, the HR was calculated by detecting the R-R intervals in the ECG.

### 6.3 Body movements and facial expressions

In this part, the body movements and facial expressions that are related to the embarrassment, interest, and surprise emotions that the participants experienced in the failure of meshing scenario (as it will be presented later) are presented.

#### 6.3.1 Embarrassment gestures

Bodily cues, and eye and mouth movements are crucial in the recognition of embarrassment (Edelmann and Hampson, 1981). Increase in body motion, reduction in eye contact (Modigliani, 1971), lowering the level of eye contact (Edelmann and Hampson, 1981), shuffling the feet and putting the finger to the lips (Birdwhistell, 1952; Krout, 1954), manipulative gestures (Schulz and Barefoot, 1974), increased smiling, disturbances in speech, and postural shifting (Edelmann and Hampson, 1981) are all cues, gestures, and signs of embarrassment. Note that, manipulative gestures are where the hand of the participant moves to touch himself although not communicating anything (Schulz and Barefoot, 1974).

Relying on facial cues only can lead to identifying embarrassment as amusement (Edelmann and Hampson, 1981). The most important signals of embarrassment are the eyes, hands, lower legs, and mouth. If the level of eye contact becomes higher, this means that the participant is amused and not embarrassed. Embarrassment can be disguised as amusement as a “face-saving” display to deceive the onlooker (Goffman, 1955). An amused expression combined with downcast eyes, nervous hands, and leg movements produces an “embarrassed laugh”.

Social anxiety and embarrassment are difficult to differentiate at the autonomic level which leads to blushing being a psychophysiological marker of social anxiety and shyness (Hofmann et al., 2006). Anxiety can be recognized by lip biting, stretching of the mouth, eyes turn up/down/left/right, lip wipe, fingers moving, and fingers tapping on a surface (e.g., table) (Gunes and Piccardi, 2007).

#### 6.3.2 Interest gestures

Interest is an emotion of a positive valence and low arousal. It can be identified by arms resting on the sides with trunk leaning forward. Asymmetrical one arm action and raising hands are also an interest emotion body movement (Castellano et al., 2008). Note that, in combination with facial expressions and the situational context, that body movement can be interpreted as a gesture that indicates either interest or irritation (cold anger) (Dael et al., 2012).

#### 6.3.3 Surprise gestures

Failure of meshing is expected to cause surprise which necessitates considering its gestures in the analysis. Surprise emotion can be recognized when the following body movements occur (Noroozi et al., 2018):

• Abrupt backward movement

• One hand or both of them move toward the head

• Moving one hand up

• Both of hands touching or over the head

• One of the hands or both touching the face or mouth

• Self-touch or both hands covering the cheeks or the mouth

• Head shaking

• Body shift or backing

## 7 Results analysis

### 7.1 Subjective ratings analysis

This is a between-subjects study with a 2 (Gender: Male, Female) × 2 (Robot Facial Appearance: Human, Anime) × 3 (Robot Personality: Neutral, Empathic, Ridiculing) design. Three-Way ANOVA was conducted to examine the effects of these three factors on various subjective emotional ratings.

#### 7.1.1 Ratings of seven emotions

**FIGURE 5:**
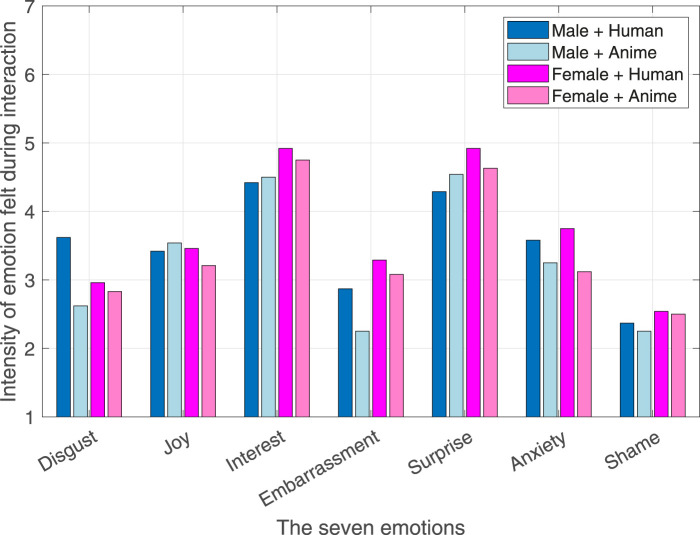
Intensity of seven emotions during the interaction.

**FIGURE 6:**
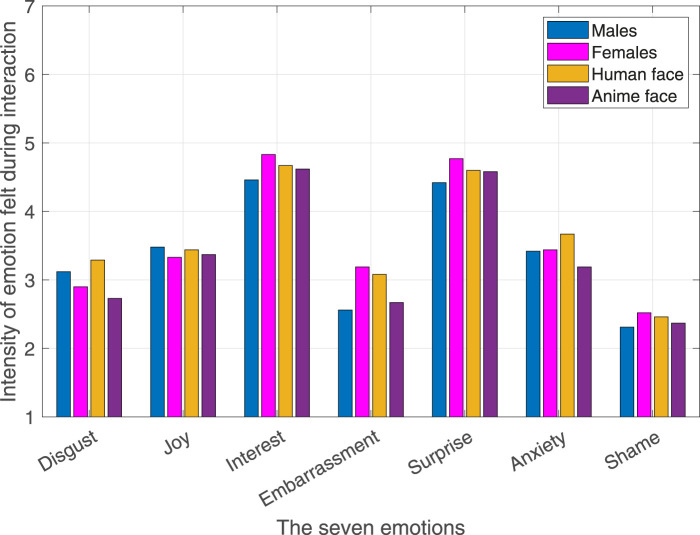
Intensity of emotions for all males, all females, all human robot face, and all anime robot face interactions.

**FIGURE 7:**
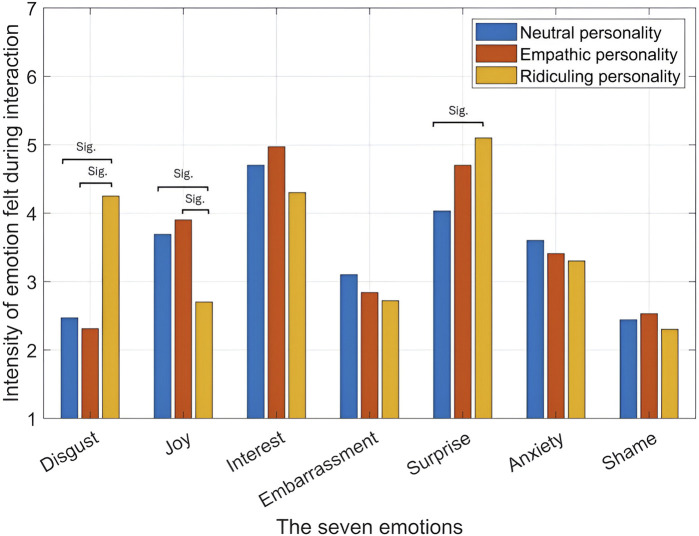
Intensity of emotions across different robot personalities.

The ratings for the seven emotions (Costa et al., 2001): disgust, joy, interest, embarrassment, surprise, anxiety, and shame are shown in Figure 5. Clearly, the emotions experienced by all the participants were primarily interest and surprise. After dividing the participants according to their gender and the robot’s social agency (facial appearance), same results were obtained as shown in Figure 6. Significant differences across the different robot personalities and the seven emotions are highlighted in Figure 7.

##### 7.1.1.1 Disgust emotion

Personality is a significant main effect, F (2,84) = 37.07, p< 0.001, η ^2^_*p*_ = 0.25. Post-hoc comparisons using Tukey’s HSD indicated that the ridiculing personality ( M = 4.25, SD = 1.92) elicited significantly higher disgust than the neutral ( M = 2.47, SD = 1.61), p< 0.001, Cohen’s d = 1.09, 95% CI [0.57, 1.62] and the empathic personalities ( M = 2.31, SD = 1.31), p< 0.001, Cohen’s d = 1.2, 95% CI [0.66, 1.72] as shown in Figure 7. This result highlights that a ridiculing response is the least favored by participants and thus, should be avoided.

##### 7.1.1.2 Joy emotion

Personality is a significant main effect, F (2,84) = 6.68, p= 0.002, η ^2^_*p*_ = 0.14. Post-hoc comparisons using Tukey’s HSD indicated that the ridiculing personality ( M = 2.66, SD = 1.47) elicited significantly lower joy than the empathic ( M = 3.88, SD = 1.5), p = 0.003, Cohen’s d = 0.848, 95% CI [0.334, 1.362] and the neutral personalities ( M = 3.69, SD = 1.26), p = 0.014, Cohen’s d = 0.72, 95% CI [0.21, 1.23] as shown in Figure 7. This result highlights that a ridiculing response is the least favored by participants and thus, should be avoided.

##### 7.1.1.3 Interest emotion

The Gender × Personality interaction was significant, F (2,84) = 5.49, p= 0.006, η ^2^_*p*_ = 0.12. Post-hoc comparisons using Tukey’s HSD indicated that males interacting with a ridiculing personality ( M = 3.81, SD = 1.8) experienced significantly less interest than females who interacted with an empathic personality ( M = 5.63, SD = 1.26), p = 0.021, Cohen’s d = 1.14, 95% CI [0.42, 1.87]. This follow-up *post hoc* comparison seems self-evident, thus, it did not yield a consistent or theoretically meaningful pattern of differences. However, it pointed out that the empathic personality ( M = 4.97, SD = 1.45) elicited the highest interest emotion over the neutral ( M = 4.69, SD = 1.51) and the ridiculing personalities ( M = 4.28, SD = 1.9) despite the differences being not significant, p = 0.22. This result highlights the potential of robot personalities to elicit interest in humans, which may influence their willingness or intention to use the robot in the future.

##### 7.1.1.4 Embarrassment emotion

There were no significant differences. However, it was observed that females ( M = 3.19, SD = 1.94) experienced higher embarrassment than males ( M = 2.56, SD = 1.62), p = 0.09. Moreover, the human face ( M = 3.08, SD = 1.91) also elicited higher embarrassment than the anime face ( M = 2.67, SD = 1.69), p = 0.27. Lastly, the neutral personality ( M = 3.06, SD = 1.83) elicited the highest embarrassment followed by the empathic personality ( M = 2.84, SD = 1.61) and then, the ridiculing personality ( M = 2.72, SD = 2), p = 0.75, as shown in Figure 7.

Despite the insignificant differences, the results show a potential for the empathic response in being a better alternative than a neutral response for embarrassment mitigation.

Note that, females experiencing embarrassment more intensely than males (Pettijohn et al., 2010; Salem and Sumi, 2024; Sato et al., 2024) and a human robot face eliciting higher embarrassment than the anime face (Bartneck et al., 2010) are both in line with the HHI and HRI literature.

##### 7.1.1.5 Surprise emotion

Personality is a significant main effect, F (2,84) = 4.13, p= 0.02, η ^2^_*p*_ = 0.09. Post-hoc comparisons using Tukey’s HSD indicated that the ridiculing personality ( M = 5.06, SD = 1.61) elicited significantly higher surprise emotion than the neutral personality ( M = 4.03, SD = 1.62), p = 0.02, Cohen’s d = 0.71, 95% CI [0.2, 1.22], as shown in Figure 7.

The Gender × Personality interaction was significant too, F (2,84) = 5.84, p= 0.004, η ^2^_*p*_ = 0.12. Post-hoc comparisons using Tukey’s HSD indicated that females who interacted with the neutral personality ( M = 3.5, SD = 1.79) experienced significantly less surprise emotion than the ones who interacted with the empathic personality ( M = 5.31, SD = 1.35), p = 0.009, Cohen’s d = 1.25, 95% CI [0.52, 1.98] and the ridiculing personality ( M = 5.5, SD = 1.41), p = 0.003, Cohen’s d = 1.38, 95% CI [0.64, 2.11].

The obtained results highlight that a minimalistic reply of confirmation where direct (as in the ridiculing response) or indirect (as in the empathic response) remarks are nonexistent can ensure the weakest surprise emotion elicitation to the participants. This is also confirmed in Figure 7 where the ridiculing response elicited the highest surprise emotion followed by the empathic response and lastly, the neutral response.

##### 7.1.1.6 Anxiety emotion

There were no significant differences. However, it was observed that females ( M = 3.44, SD = 2.08) experienced higher anxiety than males ( M = 3.42, SD = 1.75), p = 0.96. Moreover, the human face ( M = 3.67, SD = 1.78) also elicited higher anxiety than the anime face ( M = 3.19, SD = 2.03), p = 0.23. Lastly, the neutral personality ( M = 3.59, SD = 2.03) elicited the highest anxiety followed by the empathic personality ( M = 3.41, SD = 1.81) and then, the ridiculing personality ( M = 3.28, SD = 1.94), p = 0.81, as shown in Figure 7.

Despite the insignificant differences, the results show a potential for the empathic response in being a better alternative than a neutral response for embarrassment mitigation as it could elicit lower anxiety which could aid in mitigating the embarrassment experienced by humans.

Note that, as anxiety is an embarrassment-related emotion (Bartneck et al., 2010), the effects of gender and robot anthropomorphism on elicited anxiety are in line with findings from the HHI and HRI literature.

##### 7.1.1.7 Shame emotion

There were no significant differences. However, it was observed that females ( M = 2.52, SD = 1.8) experienced higher shame than males ( M = 2.31, SD = 1.5), p = 0.54. Moreover, the human face ( M = 2.46, SD = 1.62) also elicited higher shame than the anime face ( M = 2.38, SD = 1.7), p = 0.81. Lastly, the empathic personality ( M = 2.53, SD = 1.68) elicited the highest shame followed by the neutral personality ( M = 2.44, SD = 1.56) and then, the ridiculing personality ( M = 2.28, SD = 1.75), p = 0.83, as shown in Figure 7.

Although the differences were not statistically significant, the results point to a potential limitation of the empathic response: by indirectly signaling the occurrence of an embarrassing incident, it may have inadvertently increased participants’ feelings of shame compared with the neutral response. The indirect confrontation could be a reason for the empathic response having high shame rating. Nevertheless, direct confrontation occurred with the ridiculing response and did not cause high shame rating as the empathic response. It is suspected that due to the social agency of the robot, a direct confrontation was not as effective as an indirect one. This underscores a potential link between the robot’s social agency and the type of confrontation it employs in dialogue, which may significantly shape the elicitation and regulation of emotions.

Note that, as shame is an embarrassment-related emotion (Bartneck et al., 2010), the effects of gender and robot anthropomorphism on elicited shame are in line with findings from the HHI and HRI literature.

##### 7.1.1.8 Embarrassment-related emotions

Interestingly, the embarrassment-related emotions (i.e., embarrassment, anxiety, and shame) were the lowest in rating as shown in Figures 5, 6. Nevertheless, the level of perceived embarrassment following the method in Bartneck et al. (2010) where the average of the embarrassment-related emotions is used was applied.

There were no significant differences. However, it was observed that females ( M = 3.05, SD = 1.73) experienced higher embarrassment-related emotions than males ( M = 2.76, SD = 1.36), p = 0.38. Moreover, the human face ( M = 3.07, SD = 1.51) also elicited higher embarrassment-related emotions than the anime face ( M = 2.74, SD = 1.59), p = 0.32. Note that, both results are in line with the HHI and HRI literature.

The neutral personality ( M = 3.03, SD = 1.58) elicited the highest embarrassment-related emotions followed by the empathic personality ( M = 2.93, SD = 1.51) and then, the ridiculing personality ( M = 2.76, SD = 1.6), p = 0.79. This could highlight the potential of utilizing an empathic response for embarrassment mitigation despite of the possible high shame it could induce.

#### 7.1.2 Experienced embarrassment

**FIGURE 8:**
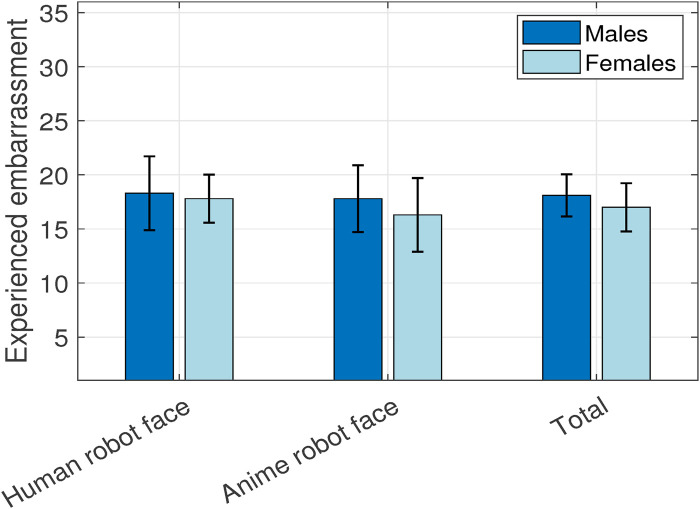
Experienced embarrassment.

The ratings obtained from administering the “experienced embarrassment questionnaire” (Modigliani, 1968) are shown in Figure 8. There were no significant differences.

Males ( M = 18.1, SD = 7.53) scored higher experienced embarrassment than females ( M = 17, SD = 6.8), p = 0.48. Human face ( M = 18.1, SD = 6.63) also elicited higher experienced embarrassment than an anime face ( M = 17, SD = 7.67), p = 0.48. Note that, both results are in line with the HHI and HRI literature.

Ridiculing personality ( M = 18.3, SD = 8.09) elicited the highest experienced embarrassment followed by the neutral personality ( M = 17.8, SD = 7.05) then the empathic personality ( M = 16.5, SD = 6.3), p = 0.6. As this questionnaire dissects embarrassment closely, the ridiculing response caused the highest embarrassment as initially expected and then followed by the neutral response and then lastly, the empathic response. Similarly to the presented results for the embarrassment, anxiety, and shame emotions, this highlights that an empathic response could be an effective alternative to the neutral response for embarrassment mitigation.

#### 7.1.3 Robot’s evaluation

**FIGURE 9:**
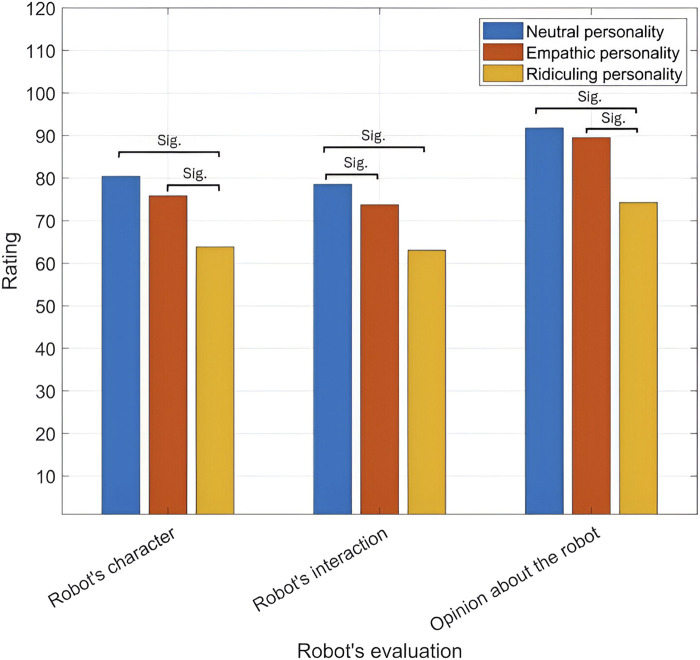
Robot’s evaluation across its three personalities.

**FIGURE 10:**
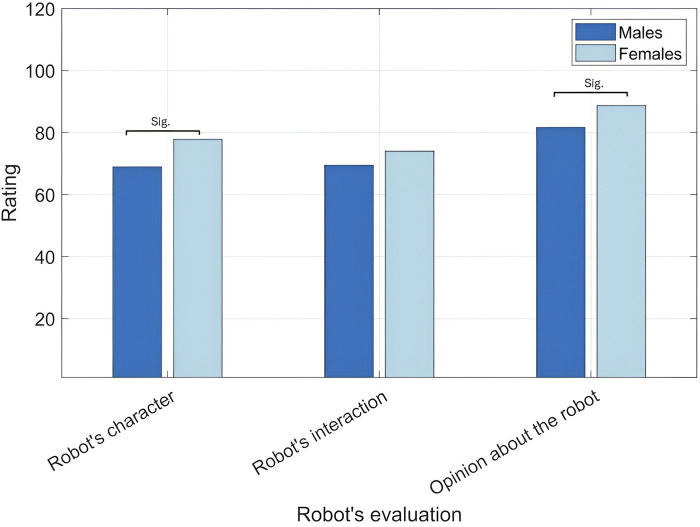
Robot’s evaluation across males and females.

The responses obtained from the three questionnaires developed in Bickmore (2003) that evaluate robot’s perceived character, the interaction with the robot, and the opinions formed about it are presented below. The robot’s evaluation across its different personalities and participants genders is shown in Figures 9, 10, respectively.

##### 7.1.3.1 The robot’s character

The robot’s perceived character for males and females across different personalities and the robot’s facial appearances is shown in Tables 6, 7, respectively. The robot’s perceived character across its different personalities and participants genders is shown in Figures 9, 10, respectively.

**TABLE 6:** Robot’s perceived character according to its personality with males and females (*M*/*SD*).

Participants gender	Robot personality			Overall
	Neutral	Empathic	Ridiculing	
Male	79.7/15.7	66.1/21.4	60.9/18.1	68.9/19.8
Female	81.1/19.3	85.5/15.7	66.7/17.6	77.8/19
Overall	80.4/17.3	75.8/21	63.8/17.8	

**TABLE 7:** Robot’s perceived character according to its social agency with males and females (M/SD).

Robot face	Participants gender		Overall
	Male	Female	
Human robot face	69.4/20	80/20.3	74.7/20.6
Anime robot face	68.4/20.1	75.6/17.9	72/19.2
Overall	68.9/19.8	77.8/19	

Participants gender is a significant main effect, F (1,84) = 5.5, p= 0.021, η ^2^_*p*_ = 0.06. Females ( M = 77.8, SD = 19) perceived the robot’s character significantly more favorably than males ( M = 68.9, SD = 19.8) as shown in Figure 10. This result highlights that females could be more accepting and trusting to robots than males.

Personality is a significant main effect, F (2,84) = 6.82, p= 0.002, η ^2^_*p*_ = 0.14. Post-hoc comparisons using Tukey’s HSD indicated that the ridiculing personality ( M = 63.8, SD = 17.8) led to perceiving the robot’s character significantly less than the neutral personality ( M = 80.4, SD = 17.3), p = 0.002, Cohen’s d = 0.9, 95% CI [0.38, 1.41] and the empathic personality ( M = 75.8, SD = 21), p = 0.031, Cohen’s d = 0.646, 95% CI [0.14, 1.15] as shown in Figure 9. This highlights that a neutral response is the most favored in terms of perceived robot character.

Lastly, it was observed that the human face ( M = 74.7, SD = 20.6) was rated higher than the anime face ( M = 72, SD = 19.2), p = 0.5. This could be due to the expressiveness of the human face compared to the anime face as it can be noticed in Supplementary Video 1.

##### 7.1.3.2 The robot’s interaction

The robot’s perceived interaction is presented in Tables 8, 9. Moreover, the robot’s interaction across its different personalities and participants genders is shown in Figures 9, 10, respectively.

**TABLE 8:** Robot’s perceived interaction according to its personality with males and females (*M*/*SD*).

Participants gender	Robot personality			Overall
	Neutral	Empathic	Ridiculing	
Male	80.1/21.3	66.6/24.6	61.6/22.9	69.4/23.9
Female	76.8/23.4	80.7/22.2	64.5/23.9	74/23.8
Overall	78.5/22.1	73.7/24.2	63/23.1	

**TABLE 9:** Robot’s perceived interaction according to its social agency with males and females (M/SD).

Robot face	Participants gender		Overall
	Male	Female	
Human robot face	71.2/26.3	76.1/25.9	73.6/25.9
Anime robot face	67.7/21.6	71.9/21.8	69.8/21.6
Overall	69.4/23.9	74/23.8	

Personality is a significant main effect, F (2,84) = 3.53, p= 0.034, η ^2^_*p*_ = 0.08. Post-hoc comparisons using Tukey’s HSD indicated that the neutral personality interaction ( M = 78.5, SD = 22.1) was perceived significantly higher than the ridiculing personality ( M = 63, SD = 23.1), p = 0.03, Cohen’s d = 0.65, 95% CI [0.14, 1.16], as shown in Figure 9. This highlights that a neutral response is the most favored in terms of perceived robot’s interaction.

It was observed that females ( M = 74, SD = 23.8) rated the interaction higher than males ( M = 69.4, SD = 23.9), p= 0.35, as shown in Figure 10. Despite of the insignificant differences, this result is similar to the former one which proves that females could be more accepting and trusting to robots than males.

The human face ( M = 73.6, SD = 25.9) received higher interaction rating than the anime face ( M = 69.8, SD = 21.6), p= 0.43, which could be due to the high expressiveness of the human face compared to the anime face as it can be noticed in Supplementary Video 1.

Lastly, the neutral personality ( M = 78.5, SD = 22.1) received the highest rating followed by the empathic personality ( M = 73.7, SD = 24.2) and then lastly, the ridiculing personality ( M = 63, SD = 23.1), p= 0.03, as shown in Figure 9. This highlights that a neutral response is the most favored in terms of perceived robot interaction.

##### 7.1.3.3 Opinion about the robot

The opinions formed about the robot are presented in Tables 10, 11. The opinions formed about the robot across its different personalities and participants genders are shown in Figures 9, 10, respectively.

**TABLE 10:** Opinion about the robot according to its personality with males and females (M/SD).

Participants gender	Robot personality			Overall
	Neutral	Empathic	Ridiculing	
Male	88.8/16.4	82.9/13.5	73.3/18.1	81.6/17.1
Female	94.7/12.5	96.1/14.6	75.3/19.9	88.7/18.3
Overall	91.8/14.7	89.5/15.4	74.3/18.7	

**TABLE 11:** Opinion about the robot according to its social agency with males and females (M/SD).

Robot face	Participants gender		Overall
	Male	Female	
Human robot face	81.4/17.3	90.8/19	86.1/18.6
Anime robot face	81.9/17.2	86.6/17.8	84.3/17.5
Overall	81.6/17.1	88.7/18.3	

Participants gender is a significant main effect, F (1,84) = 4.41, p= 0.04, η ^2^_*p*_ = 0.05. Post-hoc comparisons using Tukey’s HSD indicated that the females ( M = 88.7, SD = 18.3) formed opinions about the robot were significantly higher than males ( M = 81.6, SD = 17.1), p = 0.04, Cohen’s d = 0.43, 95% CI [0.02, 0.84], as shown in Figure 10. This result is similar to the former two results which highlights that females could be more accepting and trusting to robots than males. This evaluation was consistent along the three aspects of the robot’s evaluation.

Personality is a significant main effect, F (2,84) = 10.81, p< 0.001, η ^2^_*p*_ = 0.21. Post-hoc comparisons using Tukey’s HSD indicated that the opinions formed about the neutral personality ( M = 91.8, SD = 14.7) were significantly higher than the ridiculing personality ( M = 74.3, SD = 18.7), p< 0.001, Cohen’s d = 1.1, 95% CI [0.55, 1.6], as shown in Figure 9. Opinions formed about the empathic personality ( M = 89.5, SD = 15.4) were also significantly higher than the ridiculing personality ( M = 74.3, SD = 18.7), p = 0.001, Cohen’s d = 0.93, 95% CI [0.41, 1.45], as shown in Figure 9. Note that, the neutral personality ( M = 91.8, SD = 14.7) was rated the highest and followed by the empathic personality ( M = 89.5, SD = 15.4) and lastly, the ridiculing personality ( M = 74.3, SD = 18.7) as shown in Figure 9. This highlights that a neutral response is the most favored in terms of the opinions formed about the robot. This evaluation was consistent along the three aspects of the robot’s evaluation.

It was observed that the human face ( M = 86.1, SD = 18.6) gained better opinions than the anime face ( M = 84.3, SD = 17.5), p = 0.59, which could be due to the high expressiveness of the human face compared to the anime face as it can be noticed in Supplementary Video 1. This evaluation was consistent across the three aspects of the robot’s evaluation.

#### 7.1.4 Participants embarrassability

The participants embarrassability is shown in Table 12. The embarrassability of the males interacting with the human face (*M* = 143.87, *SD* = 22.74) was significantly higher than that of the males interacting with the anime face (*M* = 130.08, *SD* = 23.53), t (46) = 2.06, p = 0.04. There were no other significant differences.

**TABLE 12:** Embarrassability score data of the participants (*M*/*SD*).

Robot face	Participants gender		Overall
	Male	Female	
Human robot face	143.87/22.74	136.37/29.53	140.12/26.35
Anime robot face	130.08/23.53	141.87/24.45	135.98/24.48
Overall	136.98/23.93	139.12/26.96	

### 7.2 Physiological data analysis

#### 7.2.1 ECG analysis

The HR of all participants in different experiment conditions is investigated. Embarrassment leads to an increase in heart rate (Hofmann et al., 2006). As embarrassment emotion is related to the baseline (relaxation state) and the embarrassed state, the difference between the embarrassed state and the baseline state was used in the analysis. Thus, a higher difference (of positive value) can indicate that the embarrassed state’s physiological data was higher in value than the baseline state.

The robot’s face had a significant trend, F (1,82) = 3.7, p= 0.058, η ^2^_*p*_ = 0.043. Post-hoc comparisons using Tukey’s HSD indicated that the anime face ( M = 4.51, SD = 18.6) induced a significantly higher heart rate difference than the human face ( M = −2, SD = 15), p = 0.058, Cohen’s d = 0.4, 95% CI [0.014, 0.81]. It is suspected that due to the males interacting with the human face had significantly higher embarrassability and due to embarrassment occurrence in males being correlated with low HR (Salem and Sumi, 2024), the obtained result surfaced. Thus, higher embarrassment occurred with the human face leading the HR of males to be low compared to the anime face and results in making the HR difference noticeable in the sample.

It was observed that the neutral personality ( M = 4.4, SD = 17.9) elicited the highest HR difference and was followed by the empathic personality ( M = 3.47, SD = 8.58), and lastly, the ridiculing personality ( M = −3.91, SD = 21.5), p = 0.12. Although the ridiculing attitude caused the highest embarrassment and thus, the least favored, a low HR difference could be due to the negative nature of the emotion and its effect on males (Salem and Sumi, 2024). Negative emotions are associated with reduction in HR (Osnes et al., 2023). It could be also due to the high disgust elicited from the ridiculing response which is commonly known for lowering HR in males and females (Gilchrist et al., 2016; Stark et al., 2005).

#### 7.2.2 EDA analysis

During embarrassed states, arousal increases above the baseline relaxation levels, thus, similarly to the ECG study, the EDA difference is used. EDA difference is the difference between the arousal level in an embarrassed state and the baseline state. Thus, a positive high difference indicates higher arousal in the embarrassed state than the baseline state, which is an indication of an embarrassment occurrence.

There were no significant differences. However, a significant trend was observed where females ( M = 4.74, SD = 5.18) had higher arousal difference than males ( M = 2.83, SD = 4.99), p = 0.07. This result is expected and in line with the embarrassment emotion literature.

The ridiculing personality ( M = 4.96, SD = 5.53) incurred the highest arousal difference followed by the neutral personality ( M = 3.7, SD = 4.98), and lastly, the empathic personality ( M = 2.7, SD = 4.83), p = 0.22. Thus, the potential of the empathic response is highlighted again where it is possible that it can be an effective candidate for embarrassment mitigation.

The human face ( M = 3.84, SD = 5.04) induced a higher arousal difference than the anime face ( M = 3.73, SD = 5.32), p = 0.92, however, the difference was minimal. Nevertheless, this result is expected and in line with the embarrassment emotion literature.

### 7.3 Body movements, facial expressions, and participants responses

#### 7.3.1 Participants body movements and facial expressions

Two independent researchers coded the participants body movements and facial expressions. The Cohen’s Kappa was calculated to measure inter-rater reliability. A substantial agreement score of κ = 0.68 (95% CI: 0.61–0.75) was achieved (“substantial” agreement referring to 0.61 <κ< 0.80) (McHugh, 2012). Thus, raters are broadly consistent. The percent agreement was 72.5% which shows a strong agreement between the two raters. Moreover, the Gwet AC2 was 0.68 which also shows a substantial agreement between the raters (Landis and Koch, 1977; Altman, 1991) and verifies the reliability of the raters’ coding.

For further statistical analyses examining gender, robot social agency, and robot personality effects, a single consensus-coded dataset was required. Therefore, any disagreements were resolved through adjudication to ensure reliability and usability.

In the experiment, the participants were sitting in front of the robot which limited their movement. Moreover, ECG and EDA sensors were attached to them which limited their hand movements. Furthermore, looking at different paintings while holding them made their hands occupied. As a result, asymmetrical arm movements which are a body cue of experiencing interest emotion were discarded as participants hands were busy holding the paintings. Some of them showed the paintings to the robot and others just described the paintings vocally.

It is suspected that due to the constant holding of the paintings thus forcing participants to do an asymmetrical arm movement (an interest emotion body gesture) may have given them the perception that they are experiencing an emotion of interest (Davey et al., 2021; Coles et al., 2019; Kelly and Ngo Tran, 2025) which could have been rated lower if they are seeing the paintings on a screen or through any other method that does not require holding them. Any lower gazing was also not considered as the experiment setup made gazing down to be always for the purpose of looking at the paintings. Thus, gazing down was not necessarily due to embarrassment despite being a cue of it.

Note that, embarrassment emotion can be misidentified to be amusement if only facial expressions are monitored (Edelmann and Hampson, 1981). Thus, when Duchenne smile occurred with males and females, lower leg movements, hands, eyes, and mouth were also considered (Edelmann and Hampson, 1981). It was clear that in the experiment an embarrassment emotion is being experienced, not joy or amusement.

A chi-square test of independence across all the experiment’s conditions indicated that body movements and facial expressions differed significantly depending on the robot face and personality, χ (110, N = 222) = 162, p< 0.001, Cram $\overset{´}{e}$ r’s V = 0.27. Because some cells had low expected counts, Fisher’s exact test was also conducted, which confirmed the result, p = 0.002.

Post-hoc analysis of adjusted residuals revealed that manipulative gestures were significantly more frequent than expected with the human robot face with a ridiculing personality that interacts with males (adjusted residual = 2.175, p< 0.05). Manipulative gestures are cues of embarrassment occurrence. This highlights that a ridiculing robot response is highly discomforting to participants.

Smile control was also significantly more frequent than expected with the anime robot face with a ridiculing personality that interacts with females (adjusted residual = 2.528, p< 0.01). Smile control proves that embarrassment took place, however, it is possible that it occurred with the anime face due to a baby schema (Glocker et al., 2009; Yao et al., 2022) from the anime face’s big eyes. Embarrassment in front of a witness who is perceived as cute converges two social-affective processes (Keltner, 1995), which are self-conscious appraisal (as embarrassment) and an increased drive to appease or affiliate because the witness (the robot with an anime face) evokes a caregiving approach. The embarrassed smile is highly functional in this situation as it signals non-threat intent (appeasement) and manages self-presentation (modesty) which suits the situation when the actor wants to elicit care from the witness (the anime robot face).

Lip wipe was significantly more frequent with the human robot face with empathic personality that interacts with males (adjusted residual = 2.316, p< 0.05). The empathic personality is inherently kind which could trigger reciprocity (Gouldner, 1960) from humans. A form of reciprocity is through impression management where people unconsciously adjust their grooming behaviors when interacting with desirable others (Knapp et al., 1972). Subtle lip moistening can increase attractiveness as it draws attention to the mouth and also improve the appearance (Grammer et al., 2000). Lip wiping is a socially valuable tool as it signals a kind friendly persona as it is an attractiveness enhancer (Ekman and Friesen, 1969).

Duchenne smile was significantly less frequent with the anime robot face with an empathic personality that interacts with females (adjusted residual = −2.6, p< 0.01). The few occurrences of Duchenne smile with the anime robot face could be due to the fact that it is an artificial entity and the lack of its perceived agency. Non-Duchenne, polite, or social smiles are more frequent with robots than with other people (Kappas and Gratch, 2023).

Lastly, no gestures (neutral participant state were no body movements or facial expressions are taking place) were significantly more frequent with the anime robot face with a neutral personality that interacts with males (adjusted residual = −2.75, p< 0.01) and less frequent with the human robot face with a ridiculing personality that interacts with females (adjusted residual = −2.05, p< 0.05). This highlights that a neutral response is not triggering unlike the ridiculing response. Moreover, the robot’s facial appearance have a direct effect on triggering humans and eliciting emotions which was shown in this work with the lack of gestures when both, an anime face is combined with a neutral non-provoking and non-confrontational response.

To investigate the effect of gender differences and the robot’s social agency, a chi-square test of independence indicated that body movements and facial expressions differed significantly depending on the gender of the participant and the robot face, χ (30, N = 222) = 49.6, p = 0.014, Cram $\overset{´}{e}$ r’s V = 0.27. Because some cells had low expected counts, Fisher’s exact test was also conducted, which confirmed the result, p = 0.011.

Post-hoc analysis of adjusted residuals revealed that lip wipe and Duchenne smile were significantly less frequent than expected when females interact with the robot human (adjusted residual = −2.2, p< 0.05) and anime faces (adjusted residual = −2.74, p< 0.01), respectively. It can be deduced that lip wipe was less frequent with the human face robot due to absence of a need to enhance attractiveness to the robot. Less frequent Duchenne smile with the anime face robot can be due to its lack of perceived agency.

There were no significant differences between males and females regarding their body movements and facial expressions. There were also no significant differences between participants who interacted with the robot human and anime faces.

#### 7.3.2 Participants responses

Participants’ verbal responses to the robot were coded using a thematic framework corresponding to 17 predefined embarrassment remediation strategies as presented in Sections 2.1, 2.2.

There was a significant trend where remediation strategies frequency of usage differed significantly across the experiment’s conditions. Fisher’s exact test was conducted, p = 0.059.

Post-hoc analysis of adjusted residuals revealed that the apology remediation strategy was used more frequently when females interact with the robot with the human face and ridiculing personality (adjusted residual = 2.23, p< 0.05). This is in line with (Itoi et al., 1996) where Japanese females are more likely to offer an apology than Japanese males. Interestingly, males used aggression with the same condition more frequently than expected (adjusted residual = 2.1, p< 0.05) and used the “defensively changing the subject” strategy less frequently than expected (adjusted residual = −2.06, p< 0.05). Higher externalizing behaviors such as aggression or hyperactivity in response to stressors were noticed more in males than females who tend to exhibit an internalizing behavior (Ishimoto et al., 2022). Besides, traditional gender roles often encourage men to display strength and dominance, which can manifest as aggression, directness or assertiveness in communication (Ramírez et al., 2001) which makes the strategy of “defensively changing the subject” less likely to be employed by males.

Lastly, when males interact with an anime robot face with an empathic personality, the fishing for reassurance was used more frequently than expected (adjusted residual = 3.1, p< 0.05) which can be due to the baby schema or the empathic personality projected by the anime robot. Note that, for the sake of comprehensiveness, when a participant said “thank you”, it was marked as a “fishing for reassurance” strategy, however, saying “thank you” in Japanese culture is a cultural norm and expected in social interactions (Ohashi, 2008).

Fisher’s exact test also confirmed gender differences in using different remediation strategies, p = 0.003. However, there were no significant differences in the chosen remediation strategy for both robot faces.

Across all conditions, the justification strategy was the most used strategy (22.43%) followed by the strategy of defensively changing the subject (19.2%), accept failure (10.2%), do nothing (8.3%), minimizing failure by derogating the task (7.05%), introducing redeeming or self-enhancing information (6.4%), introducing information excusing performance (5.8%), fishing for reassurance (5.8%), apology (4.5%), verbal aggression (2.6%), humor (2.6%), excuse (1.9%), remediation (1.3%), avoidance (0.64%), statement of the fact (0.64%), denying failure (0.64%), and lastly straightforward answers (0%).

**FIGURE 11:**
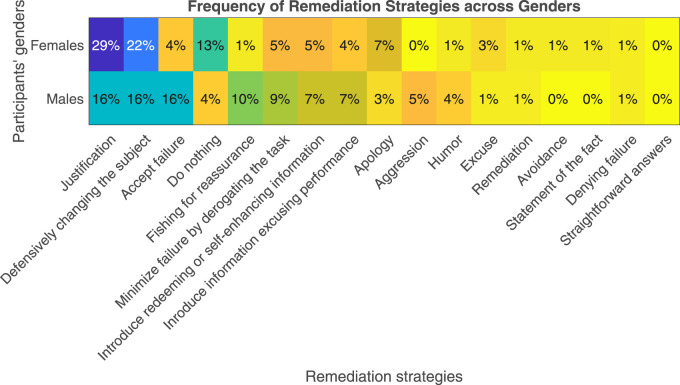
Frequency of remediation strategies across males and females.

**FIGURE 12:**
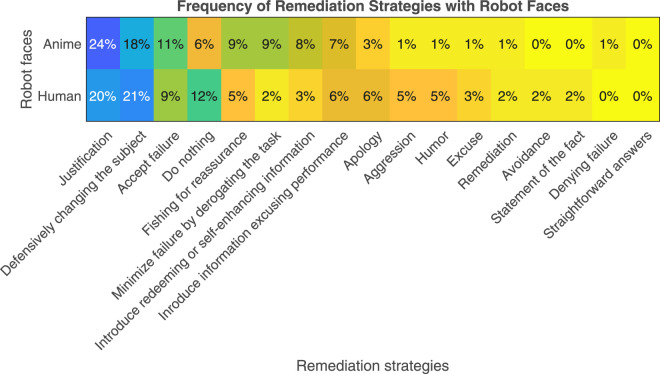
Frequency of remediation strategies across the robot’s human and anime faces.

Gender and robot facial appearance effects on the participants’ choice of the remediation strategy are shown in Figures 11, 12, respectively. Across both figures, the remediation strategies most commonly applied are the “justification” and “defensively changing the subject”. Clearly, no changes took place due to different genders or robot facial appearances.

## 8 Discussion

In this experiment, participants subjective ratings showed that they did not experience embarrassment noticeably. Moreover, interest and surprise emotions were rated highly while other embarrassment-related emotions such as embarrassment, anxiety and shame were rated low. Despite the differences not being statistically significant, embarrassment-related emotions showed a consistent trend: they were rated higher for female than for male participants, which is in line with gender differences reported in the embarrassment emotion literature (Pettijohn et al., 2010; Salem and Sumi, 2024; Sato et al., 2024). Moreover, the embarrassment-related emotions were also rated higher for the robot human face than the anime face, which is in line with the robot anthropomorphization effect on the embarrassment experienced by the participants (Bartneck et al., 2010). It is possible that embarrassment-related emotions were not rated highly as embarrassment was not experienced consciously due to its short-lived momentarily nature in a failure of meshing scenario. This is plausible since embarrassment surfaced in other subconscious physiological, body movements, facial expressions and verbal responses measures.

For emotions subjective ratings, robot personality was the main significant effect. This highlights that robot personality can have a more prominent role than the robot’s social agency or the gender of the participants in emotion regulation and alleviation scenarios where emotion control is of interest. Interestingly, the induced embarrassment and anxiety emotions were rated the highest for the neutral personality unlike the ridiculing personality where they were rated the lowest. It is suspected that the surprising nature of the ridiculing response lead to this result and aided in making the participants be more surprised than being embarrassed, thus, being distracted from the fact of how embarrassing it is (Chan and Singhal, 2013). Note that, the ridiculing personality significantly induced the highest surprise emotion. However, the experienced embarrassment questionnaire uncovered how embarrassing the ridiculing response is for participants. It was also observed that the empathic personality induced the highest shame emotion over the neutral and ridiculing personalities. This could be due to highlighting the fact that the participant gave a wrong answer in the robot’s response which could be perceived as confrontational (despite being indirect) which is not favored in Japanese culture (Brett et al., 2014). The results highlighted that an empathic response have the potential to be an effective alternative to a neutral response for embarrassment mitigation. Nevertheless, an empathically indirect confrontational response risks eliciting shame emotions (an embarrassment-related emotion). Besides, the empathic response elicited the lowest experienced embarrassment, thus, a thorough study could be beneficial to uncover its potential in emotion control.

A thorough robot evaluation showed that the robot’s perceived character, interaction, and opinions about the robot are affected by the robot’s social agency and the participants gender. The ridiculing personality received the lowest ratings whereas the neutral personality was highly favored. Interestingly, females gave the robot higher ratings than males across all aspects, including its character, interaction quality, and the opinions formed about it. Thus, when designing the robot to handle failure of meshing situations, females are easier to perceive the robot positively despite of the momentarily short-lived elicited embarrassment. This is in line with the literature where females are known to report higher trust in robots than males (Gallimore et al., 2019). Females are also more willing than males to self-disclose to robots (Uchida et al., 2020), which may explain why they tend to accept robots more easily.

Embarrassment emotion leads to an increase in heart rate and arousal. Thus, we analyze the physiological data of the participants to recognize and verify the existence of the embarrassment emotion through its psychophysiological correlates. Emotion control was realized through the robot success in mitigating and amplifying the embarrassment emotion in the participants through its responses. The heart rate when interacting with the anime face was higher than when interacting with the human face. In Salem and Sumi (2024), it is suggested that when males experience embarrassment, their heart rate decreases. Moreover, the embarrassability of males interacting with the human robot face is significantly more than the males interacting with the anime robot face. This highlights the possibility of the human face inducing more embarrassment than the anime face despite of the higher HR for the anime face. EDA results confirmed the elicitation of embarrassment emotions where females EDA was higher than males, and human robot face induced higher EDA than the anime robot face which is in line with the literature. Lastly, the ridiculing personality induced the highest EDA over other personalities. This shows a favoring for robots with an anime face and a neutral or an empathic personality for failure of meshing scenarios. Note that, an empathic response elicited the lowest EDA.

Participants responses showed that embarrassment remediation strategies are applied even with robots. Thus, the knowledge accumulated regarding embarrassment remediation strategies in HHI are applicable to the HRI field. Participants using remediation strategies with robots show that HHI social norms are being maintained in HRI contexts. Results show that many participants used more than one strategy at a time. Interestingly, participants perceived the robot with the human or anime face as not low in status, which lead them to use embarrassment remediation strategies and facework to improve their image in front of the robot (although not always due to the usage of the “do nothing” remediation strategy). The robot’s facial appearance nor the participant’s gender affected the type of remediation strategy used. Moreover, the “justification” strategy was the one mostly used followed by “defensively changing the subject” which confirms that embarrassment has been experienced due to the scenario and is being remedied. It is worthwhile to notice that strategies such as “apology” and “excuse” were almost rarely used which verifies that participants did not view the robot as higher in status than them, thus, it is not a superior-subordinate relationship, which is in line with the embarrassment remediation strategies used in Japanese culture.

It should be highlighted that some participants responses might be analyzed differently. In our experiment, all the participants are of a Japanese ethnicity. In Japanese culture, saying *“Thank you.”* and *“I do not mind.”* is common and can be considered as a social norm (Haugh and Obana, 2011). These responses were interpreted as “fishing for reassurance” type of remediation strategy to provide a comprehensive analysis. However, it is possible that, because such responses are a social norm, the motive behind them is not actually fishing for reassurance. Thus, cultural background is essential when analyzing the responses.

By observing the robot gestures, it was clear that the human face is more expressive than the anime face. The big eyes of the anime face limited the eye movements highly unlike the human face where eyes are more flexible to express various emotions. Furthermore, the anime face mouth opening was narrow which limited the expressions and consequently the emotions it can transmit unlike the human face’s mouth. It is possible that limiting the expressiveness of robot’s face during negative emotions situations could be an elegant solution that aids in mitigating negative emotions. Moreover, the big eyes of the anime face could have projected a baby schema which is usually perceived to be cuter than small eyes (of the human face) (Glocker et al., 2009; Yao et al., 2022), thus leading participants to be less embarrassed and feeling calmer (less arousal, lower heart rate, and softer and more accepting responses).

## 9 Limitations

The anime robot face was not as expressive as the human robot face. On one hand, this gave an indication that limiting the expressiveness of the robot face can aid highly in limiting the effect and contagiousness of negative emotions. On the other hand, testing the social agency could be questionable due to the dissimilarity between both faces in expressing emotions. Thus, an anime face with similar facial expressions to the human face would give a clearer indication about the effect of social agency in mitigating embarrassment.

The social robot Furhat is a back-projected robotic head which lacks a body. Thus, the embarrassment in this experiment occurred solely from the robotic head. It can be expected that more embarrassment will be experienced if the robot is having a full body and utilizing arms and hands gestures. Thus, this work gives a conclusive result for advanced back-projected robotic heads (e.g., Furhat), not robots that has a full body (e.g., humanoids).

A perception-based manipulation check was not conducted due to the short-lived nature of the elicited embarrassment from a failure of meshing scenario. However, a perception-based manipulation check would aid in ensuring that the different robot attitudes were perceived as intended which can enhance the generalizability of the results. A manipulation check prior to the main experiment would add an additional layer that validates the clarity and effectiveness of the attitude manipulation.

## 10 Conclusion

As LLMs become increasingly integrated with robots, the likelihood of embarrassing situations emerging in HRI increases. One such scenario is failure of meshing, where a misalignment in expectations leads to a short-lived but socially significant embarrassment experience. Embarrassment can negatively affect individuals, particularly shy and socially anxious people, making its regulation an important aspect of smooth and comfortable HRI.

In this study, robots methods in mitigating embarrassment through their responses were investigated through analyzing participants’ subjective ratings, physiological data, body movements, facial expressions, and verbal responses. The obtained results confirm that robot responses can successfully modulate embarrassment, either by mitigating or amplifying it. The robot’s social agency played a crucial role in shaping participants’ physiological and behavioral responses. The anime-faced robot elicited milder and softer emotional responses, resulting in lower arousal and better user ratings. This highlights the importance of social agency in mitigating embarrassment experiences.

Furthermore, it was observed that robot attitude had a significant effect on perceptions about the robot. Neutral and empathic attitudes were rated the most favorable as they facilitated smoother interactions and helped mitigate embarrassment more effectively. Interestingly, an empathic robot personality is suspected to be the most effective in embarrassment mitigation while carrying the risk of indirectly shaming the participant through its inherently indirect confrontational approach which could lead to reducing its effectiveness in mitigating embarrassment emotions. However, other embarrassment-related emotions (embarrassment and anxiety) were rated the lowest for the empathic response. Robot personality and attitude shaped user perceptions thus reinforcing the idea that emotional intelligence in robots depends on how they respond to social and emotional cues rather than their anthropomorphism alone.

## 11 Implications and future directions

Our findings contribute to the growing field of emotion regulation in HRI, demonstrating that robots can actively modulate user emotions rather than merely transmitting them. As conversational AI continues to advance, designing robots that can strategically regulate emotions, particularly embarrassment, will be crucial for ensuring more natural and comfortable interactions.

Future research should explore:

- The long-term effects of robot-mediated emotion regulation on user trust and engagement.
- Cross-cultural differences in embarrassment perception and regulation in HRI.
- How different levels of robot expressiveness impact emotion regulation outcomes.
- Potential applications in fields such as mental health, customer service, and social assistance, where emotion regulation plays a crucial role.

By advancing our understanding of how robots can recognize, respond to, and regulate emotions like embarrassment, this study lays the groundwork for more emotionally intelligent robots capable of fostering positive human-robot interactions in everyday life.

## Data Availability

The original contributions presented in the study are included in the article/Supplementary Material, further inquiries can be directed to the corresponding author.
